# Carbon Dots as an Emergent Class of Antimicrobial Agents

**DOI:** 10.3390/nano11081877

**Published:** 2021-07-22

**Authors:** Mattia Ghirardello, Javier Ramos-Soriano, M. Carmen Galan

**Affiliations:** 1 School of Chemistry, University of Bristol, Cantock’s Close, Bristol BS8 1TS, UK; 2 Centro de Investigaciones Científicas Isla de La Cartuja, Glycosystems Laboratory, Instituto de Investigaciones Químicas (IIQ), CSIC and Universidad de Sevilla, Américo Vespucio 49, 41092 Sevilla, Spain; javiramossoriano@gmail.com

**Keywords:** carbon dots, antibacterial, biofilm, drug delivery, CDs composites, bacterial sensing, wound healing, antimicrobial agents

## Abstract

Antimicrobial resistance is a recognized global challenge. Tools for bacterial detection can combat antimicrobial resistance by facilitating evidence-based antibiotic prescribing, thus avoiding their overprescription, which contributes to the spread of resistance. Unfortunately, traditional culture-based identification methods take at least a day, while emerging alternatives are limited by high cost and a requirement for skilled operators. Moreover, photodynamic inactivation of bacteria promoted by photosensitisers could be considered as one of the most promising strategies in the fight against multidrug resistance pathogens. In this context, carbon dots (CDs) have been identified as a promising class of photosensitiser nanomaterials for the specific detection and inactivation of different bacterial species. CDs possess exceptional and tuneable chemical and photoelectric properties that make them excellent candidates for antibacterial theranostic applications, such as great chemical stability, high water solubility, low toxicity and excellent biocompatibility. In this review, we will summarize the most recent advances on the use of CDs as antimicrobial agents, including the most commonly used methodologies for CD and CD/composites syntheses and their antibacterial properties in both in vitro and in vivo models developed in the last 3 years.

## 1. Introduction

Antimicrobial resistance (AMR) has become a major threat that affects public health. One of the main causes for this problem is the extensive and disproportionate use of antimicrobial agents, which has led to the selection of drug-resistant pathogens that have developed new resistance mechanisms. The fast evolution of new AMR machineries, combined to the slow development and low approval rate of new drugs, has resulted in a major global health crisis [1,2]. Multidrug resistant bacteria isolated in hospitals represent an increasing risk factor especially for surgery and intensive care unit patients [3], making it harder, if not impossible, to treat infections with the consequent increase of medical complications and sanitation costs [4,5].

The development of reliable, cheap, and fast strategies for determining the presence or absence of bacteria or identification of specific species/strains in patient samples could reduce inappropriate prescribing of antibiotics in primary and secondary care [6]. Similarly, the ability to target antimicrobials to specific pathogens could reduce the inappropriate use of broad-spectrum antibiotics, which, as mentioned earlier, drives the emergence of both antibiotic resistance and healthcare-associated infections [7]. Lastly, the targeted delivery of antibiotics directly to the surface of specific cells may enhance their antibacterial activity through increasing local concentration or stimulating intracellular uptake [8]. The issue of antibiotic targeting is of particular relevance when considering Gram-negative bacteria, as the outer membrane of these species presents a formidable barrier to ingress for many antibiotic classes, which reduces treatment options for these organisms and complicates the development of new antibiotics [9]. This is exemplified by the fact that few new agents effective against Gram-negative bacteria are currently in clinical development. For example, oxazolidinones represent the first new chemical class of antibiotic to reach the clinic in over 30 years. These molecules are inhibitors of bacterial protein biosynthesis and represent an important class of drugs that are effective against a range of Gram-positive bacteria including multiresistant pathogens such as methicillin-resistant *Staphylococcus aureus* (MRSA). However, linezolid, the first such agent to reach the clinic, is becoming compromised by the emergence of resistance [10]. Thus, the prevention of infections through the early detection of pathogens and the development of new antibacterial agents able to circumvent bacterial multidrug resistance (MDR) represent a crucial objective of current biomedical research.

One of the most promising strategies in the fight against MDR pathogens entails the photodynamic inactivation of bacteria promoted by photosensitisers. This strategy relies on the light-promoted generation of highly reactive oxygen species (ROS) able to inactivate bacterial cells in different ways, including membrane destruction and/or irreversible protein and DNA damage [11,12,13]. The most important feature of photodynamic therapies consists of the generation of a closely localized physicochemical environment that is harsh to bacterial cells in ways such as ROS production or temperature increases (known as photothermal (PTT) effects), against which it is difficult to generate a resistance [14,15]. Among the different kind of photosensitisers, carbon dots (CDs) have emerged as a promising class of nanomaterials for the specific detection and inactivation of different bacterial species [16,17]. CDs are a class of quasispherical carbon-based fluorescent nanomaterials with a typical size of 10 nm or below. These materials possess great chemical stability, high water solubility, and outstanding photoelectric properties. In addition, they exhibit low toxicity and excellent biocompatibility [18]. These features, together with their ease of preparation and reduce material costs, makes CDs ideal candidates for antibacterial theranostic applications. Indeed, since their serendipitous discovery in 2004 by Xu et al. [19], CDs have found further applications across many scientific disciplines including semiconductors [20,21], biomedicine [22,23] catalysis [24], sensing and functional materials [25,26], and in the agricultural field [27,28].

In this review, we aim to summarize the most common synthetic methodologies for accessing CD and CD/composites and their application for bacterial detection, as well as their bactericidal properties in both in vitro and in vivo models developed within the last 3 years.

## 2. General Synthetic Strategies for CDs Preparation

**Figure 1:**
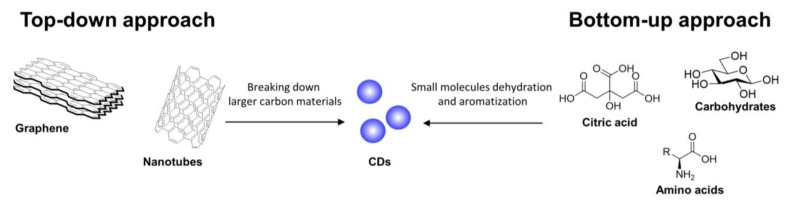
Schematic representation of top-down and bottom-up synthetic approaches for the preparation of CDs.

There are two main routes to synthesize CDs, namely top-down and bottom-up approaches [29]. CDs can be prepared starting from a plethora of different precursors, including carbon-based materials, polymers, and biomass, among others (depending on the selected approach [30,31,32]). In top-down approaches, large-sized carbon materials such as carbon nanotubes and graphite are treated under different conditions such as laser ablation, oxidative cleavage, hydrothermal, solvochemical, microwave, and ultrasonic assisted processes to allow for the generation of nanoparticles (Figure 1). Moreover, complex systems such as industrial waste, plant, fungi, and bacterial derivatives that do not present large polyaromatic structures can be decomposed under thermal conditions undergoing a cascade of dehydration and carbonization events leading to a polyaromatization process and, eventually, the formation of the CD core. On the other hand, bottom-up approaches rely on small molecules and polymers as carbon precursors for the formation of CDs (Figure 1).

Different methodologies can be used for the thermal decomposition of the starting materials, including reflux under acidic or basic conditions, hydrothermal treatment, chemical oxidation, and ultrasonic or microwave-assisted syntheses. Unlike top-down approaches that require pre-existing aromatic structures, in the bottom-up approaches almost any organic material susceptible to thermal decomposition can be used under thermal conditions for the preparation of CDs.

Within the context of bacteria-targeting CDs that will be discussed in this review, bottom-up synthetic approaches constitute by far the major synthetic strategy adopted [20]. In these synthetic studies, small organic molecules, amino acids and other natural starting materials (e.g., sugars) are utilized for the synthesis of CDs via hydro/solvochemical decomposition. It is worth noting that among the different carbon sources available for CD bottom-up syntheses, citric acid is a popular choice as a CD precursor. Moreover, the incorporation of heteroatoms (N, P, and S, among others) in the CD structure highly improves the fluorescent properties of these nanomaterials, enhancing the fluorescent quantum yield and influencing the absorbance and emission band toward a blue or red shift [33]. Even though the origin of the CD fluorescence is still under discussion, there is a common agreement that fluorescence properties are linked to CD size, surface defects, and functional groups and oxidation state [34,35,36]. Furthermore, CDs can be excited by light energy, which generates a charge separation and the formation of electrons and hole pairs trapped on the CD’s irregular surface, generating an exited state that decays via fluorescent emission to promote ROS formation. Synthetic methods for accessing CDs have been previously reviewed [29,33,37,38,39,40] and as such will not be covered in detail within this perspective.

## 3. General Antibacterial Mechanisms of Action of CDs

The CD’s functional and biological properties are directly linked to the nanomaterial core structure and, in particular, to the functional groups exposed on the CD surface. Moreover, CD structure is strictly dependent on the choice of precursors and synthetic methods adopted during the manufacturing process. Thus, small differences in terms of type of precursors, solvents, and synthetic approaches lead to the formation of structurally different nanoparticles. This makes it difficult to predict the potential antibacterial efficacy and specificity for any novel CD without undertaking exhaustive structural characterization studies. In this section, a brief summary of the most relevant examples of synthetic CDs and their interaction with the bacterial cell surface will be highlighted. For a more comprehensive description of CD’s antibacterial mechanism of action see current reviews [41,42,43,44].

**Figure 2:**
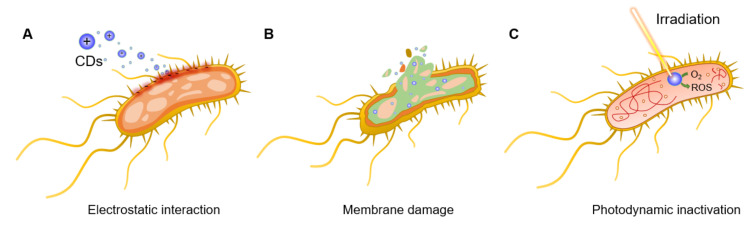
General bactericidal mechanisms of action of CDs. (**A**) Schematic representation of the initial electrostatic interaction between CDs and the bacterial cell wall. (**B**) CDs internalization, intercalation in the bacterial membrane, and irreversible disruption with a leak of cytoplasmatic material. (**C**) CD-promoted bacterial photodynamic inactivation with ROS production and DNA damage.

For instance, Bing et al. [45] demonstrated that the surface charges of CDs play a pivotal role in the initial interaction with bacterial species and, thus, in their fluorescent labelling. Positively charged CDs interact via electrostatic interactions with the negatively charged *Escherichia coli* (*E. coli*) cell wall, promoting nanoparticle internalization and bacterial apoptosis (Figure 2A). This initial interaction step is achieved thanks to the presence of ammonium on the surface of CDs and carboxyl and phosphate salts on the bacterial cell wall [46]. The bacteriostatic or bactericidal effect of CDs can be exerted through several major routes, including physical and mechanical damage to the bacterial membrane, destruction of bacterial cell wall with consequent leakage of cytoplasmic material (Figure 2B) [47], inactivation via PTT effects due to localized temperature increase [15], direct or light promoted generation of ROS [48,49], and DNA and protein damage and fragmentation (Figure 2C) [50]. Bacterial inactivation promoted by membrane damage is also a commonly observed consequence of CD intercalation in the bacterial membrane [45,51]. Additionally, N-doped CDs can be used as photosensitizers for the generation of ROS under UV or visible light irradiation, which leads to bacterial oxidative stress via the formation of H_2_O_2_, hydroxyl radicals (·OH), a superoxide anion (·O_2_^−^), and singlet oxygen (^1^O_2_) upon reaction with water and dissolved O_2_. PTT effects using near infrared (NIR) laser absorption are often exploited as a mechanism to activate the nanoparticles and induce a localized increase in temperature with consequential bacterial death. To this purpose, CDs doped with transition metals within their core structure (or via the formation of CD/metal nanoparticle composites) have been shown to be effective strategies to increase NIR PTT effects [52]. Finally, positively charged CDs are also able to bind bacterial DNA and RNA molecules, leading to the fragmentation of the genetic material and subsequent cellular inactivation, as demonstrated in the inactivation of *E. coli* and *Staphylococcus aureus* (*S. aureus*) models [53].

## 4. CDs as Bacteria Targeting and Antibacterial Agents

As already mentioned in the previous section, CDs antibacterial activity strongly correlates with the N content of CDs and surface charges. Thus, many reported antimicrobial CDs use amines or quaternary ammonium salts as starting materials for the synthesis of these carbon-based probes to ensure the formation of CDs with cationic groups on the CD surface [51,54,55,56,57,58,59,60].

Nitrogen (as well as different heteroatoms such as P, O, and S) has also been incorporated during the CD synthesis using a mixture of different starting materials [61]. For example, the addition of polyamines [16,62], amino acids [63,64,65,66,67], or biomass such as plant leaves and fungi [68,69,70,71,72,73,74], food [75,76,77,78,79], and industrial [80] and waste derivatives [81,82], among others, have been used for the preparation of doped CDs. In this context, two independent comparative studies from the Kang [83] and Bandosz [84] research groups highlighted that N- or S-doped CDs exhibit very different antibacterial properties. The authors demonstrated that the increased S-doping content correlates with an increase in repulsive forces between the less positively charged CDs (when compared to N-doped CDs) and the negatively charged bacterial surfaces. Moreover, the formation of a different bandgap between the CDs’ fundamental and exited state resulted in diminished ROS formation and, in turn, reduced bactericidal action. In a further example reported by Zhang et al. [48], the correlation between the photo-oxidative activity of CDs and the CDs’ phosphorescent quantum yield and N content was established, highlighting the importance of N-doping to increase the CDs’ photosensitization performance. On the other hand, Marković et al. [85] showed that F- and Cl-doped CDs reduce the photodynamic antibacterial effect of the nanoparticles as compared to nondoped ones, as a result of a reduced ROS production. The collective analysis of these data, points to the fundamental importance of N-doping during the CD synthesis as an essential feature to generate CDs with good antibacterial activity. 

Within the next sections, we will illustrate the most recent advances in the use of heteroatom-doped CDs as antibacterial agents, for biofilm eradication and drug delivery applications.

### 4.1. Applications of CDs as Labelling and Bactericidal Agents (Theranostics)

In many recent examples CDs found successful applications in both the labelling and eradication of different bacterial species including both Gram-positive and Gram-negative models. Among the different characterization techniques, fluorescence microscopy and ζ-potential analysis constitute the preferred methods to determine the effective interaction, labelling, and internalization of CDs with bacteria. In spite of the difference in the cell wall composition of Gram-positive and Gram-negative bacteria, they all present an overall negative charge at the bacterial surface that promotes the initial electrostatic interaction with positively charged CDs. The effectiveness of CDs as antibacterial agents is typically determined via a series of techniques such as a disk diffusion assay, determination of the bacterial optical density (OD), and colony-forming unit (CFU) counting, among others. Finally, scanning electron microscopy (SEM), detection of ROS production, and proteomic analysis, represent the most frequently used techniques for the determination of the mechanism of action of CDs. It is important to remark that small changes in the CD precursors and synthetic approaches will lead to different king of nanoparticles. This may result in substantial changes in the CDs’ bacterial labelling specificity and bactericidal activity, making impossible to predict if a novel type of nanoparticle will selectively interact with Gram-positive or Gram-negative bacterial species. Therefore, a comprehensive use of the aforementioned techniques is required to unveil the CDs’ antibacterial mechanism of action and specificity toward different bacterial species. 

**Figure 3:**
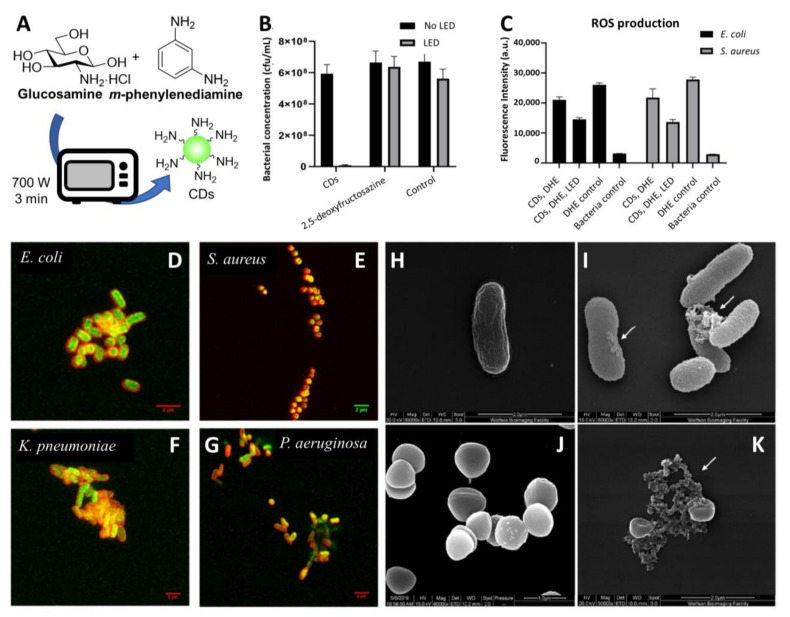
(**A**) General synthetic approach for the microwave synthesis of green fluorescent CDs. (**B**) Antimicrobial effects of combined CDs treatment and LED irradiation. Viable count shown as CFU/mL (colony forming units/mL) of *E. coli* cells treated with green CDs (200 μg/mL), commercial 2,5-deoxyfructosazine (30 μg/mL) and controls with and without 90 min LED irradiation. (**C**) CD treatment and LED illumination showing ROS production in Gram-positive and Gram-negative bacteria as demonstrated by the decrease of fluorescence intensity at 460 nm (DHE emission) for *E. coli* and *S. aureus* incubated with 200 μg/mL CDs for 90 min. (**D**–**G**) Confocal images of different bacterial species incubated with membrane dye FM 4–64 (red) and CDs (green). (**H**,**I**) Representative SEM images of *E. coli* treated with CDs in absence of LED light. (**J**,**K**) Representative SEM images of *S. aureus* treated with CDs in absence of LED light. Reproduced from a bioRxiv preprint server [86].

Recently, Galan and coworkers reported the synthesis of novel 2,5-deoxyfructosazine-coated green fluorescent CDs able to label both Gram-positive (*Staphylococcus aureus*) and Gram-negative (*Escherichia coli* (*E. coli*), *Klebsiella pneumonia* (*K. pneumonia*), and *Pseudomonas aeruginosa* (*P. aeruginosa*)) bacterial species [86]. The use of a green-fluorescent reporter improved the labelling sensitivity owing to a decreased overlap between the CDs emission band and the natural autofluorescence emission of the microorganisms. The CDs were synthesized in one pot from *m*-phenylenediamine and glucosamine·HCl as precursors in a domestic microwave (Figure 3A) [87]. The group demonstrated that the CDs have a photosensitiser effect under irradiation with blue-light emitting diodes (LEDs, λ_em_ = 460 nm). The treatment of bacterial cells with CDs at 200 µg/mL and 4 h of irradiation led to complete bacteria killing, while no significant antibacterial activity was detected in the absence of LED light or by the surface molecule 2,5-deoxyfructosazine in isolation, demonstrating that the functionalised CD is key for the activity (Figure 3B). Further studies using the superoxide indicator dihydroethidium (DHE) suggested that ROS are elicited upon irradiation of CDs with LED light in both Gram-positive and Gram-negative bacterial models (Figure 3C). 

Confocal microscopy images of bacteria incubated with green fluorescent CDs and stained with red membrane dye showed that the nanoparticles were internalized in all of the four bacterial species (Figure 3D–G). SEM imaging revealed clear signs of irreversible cell wall damage with a leak of cytoplasmatic material on both *E. coli* and *S. aureus* cells after incubation with CDs even in the absence of LED light (Figure 3H–K). Moreover, a mild photothermal effect was detected after CD-treated bacteria were exposed to LED irradiation without the need of CD doping with transition metals. Finally, tandem mass tag proteomic (TMT) analysis on *E. coli* and *S. aureus* treated with CDs and LED irradiation found changes in protein expression in both bacterial species. These findings are in agreement with other studies by Zhao et al. [88], who demonstrated that the CD’s antibacterial effects are a cumulative result of DNA and RNA damage, ribosomal transcription, oxidative stress, and membrane damage-related toxicity.

Similar results were reported by Li et al. [53], where the synthesis of vitamin C-derived CDs via electrochemical method with a broad antibacterial and antifungal activity are described. These CDs were able to inhibit the growth of both Gram-positive (*S. aureus* and *Bacillus subtilis* (*B. subtilis*)) and Gram-negative (*Bacillus* sp. *WL-6* and *E. coli*) bacteria at a range of concentrations with complete growth inhibition for all tested species at 100 µg/mL. The study showed that the antibacterial effect was exerted through the combination of membrane and DNA damage, which was caused by the ability of the CDs to bind both RNA and DNA, disrupting the DNA folding.

**Figure 4:**
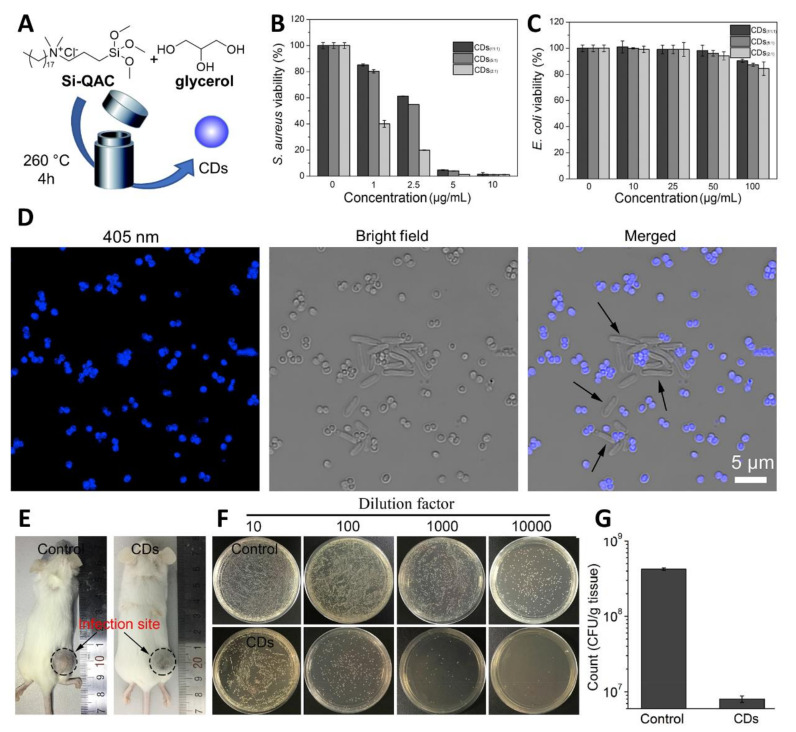
(**A**) General synthetic approach for the solvothermal synthesis of fluorescent CDs. (**B**,**C**) Cell viability evaluation results of *S. aureus* and *E. coli* after treatment with different concentrations of CDs. (**D**) Confocal images of *E. coli* and *S. aureus* cell mixtures after incubation with CDs (5 μg/mL) for 1 h (excitation at 405 nm). (**E**) Typical photographs of *S. aureus*-infected mice treated with PBS (control) or CD suspension. (**F**) Photographs and (**G**) statistical histogram of bacterial colonies counted from the dilutions of homogenized infected tissues. Reproduced with the permission from Elsevier [91].

Many current examples in the literature of antimicrobial CDs exhibit broad antimicrobial activity against MDR bacteria (such as those made from citric acid [89] and protamine sulfate [90]). Whereas this feature may be useful for environmental and surface sanitation purposes, high selectivity towards a specific bacteria target is desired for in vivo applications to avoid off-target effects. In this context, Wu and coworkers reported the synthesis of CDs with excellent labelling selectivity toward Gram-positive bacteria and in vivo antibacterial activity [91]. The nanoparticles were prepared via solvothermal synthesis from glycerol and the quaternary ammonium salt dimethyloctadecyl [3-(trimethoxysilyl)propyl]-ammonium chloride (Si-QAC) using different reagent ratios (Figure 4A). The CDs showed great selectivity for Gram-positive *S. aureus* cells with significant killing observed at concentrations as low as 5–10 μg/mL (Figure 4B), with negligible effects on Gram-negative *E. coli* (Figure 4C). The specific bacterial killing effect was further demonstrated via SEM imaging showing clear signs of membrane disruption and cytoplasmatic material leakage for *S. aureus*, while *E. coli* cell remained intact after CD treatment.

Confocal microscopy images of CD-treated bacteria provided further evidence on the staining selectivity for *S. aureus* over *E. coli* in a mixture containing both bacterial species (Figure 4D), where only *S. aureus* were stained by the blue-fluorescent CDs. The authors further expanded the pool of bacterial species showing good staining performance in Gram-positive bacterial species such as *S. aureus*, *B. subtilis,* and *Micrococcus luteus*, while negligible fluorescence was recorded for Gram-negative bacteria including *E. coli*, *Proteus vulgaris,* and *P. aeruginosa*. The team then demonstrated good biocompatibility and effective antibacterial properties of CDs in the treatment of mice infected with *S. aureus*, detecting a great CFU reduction in the infected sites compared to untreated mice (Figure 4E–G). 

Similarly, Wang et al. [92] reported how tartaric acid-based CDs prepared via hydrothermal methods bear a strong positive charge on their surface and can selectively interact with and kill Gram-positive *S. aureus* with minimal antibacterial effect on the Gram-negative *E. coli* model. The authors correlated the CD’s selectivity toward Gram-positive bacteria to the different composition of the bacterial cell walls. On one hand, the complex Gram-negative cell wall is made of multiple layers of peptidoglycans, lipoproteins, phospholipids, and lipopolysaccharides, which hampers the penetration of the CDs in bacterial cells. On the other side, the higher negative charge density present on Gram-positive bacteria compared to Gram-negative ones promotes the electrostatic interaction between CDs and the bacterial cells directing the CDs specificity toward Gram-positive species [93]. Collectively, these data demonstrate the potential of CDs as theranostic agents.

### 4.2. Applications of CDs for Biofilm Eradication and Inhibition

Bacterial biofilm is a microenvironment generated by most bacterial species to protect themselves from external agents (such as adverse environmental and biochemical conditions) that leads to an increased bacterial survival rate [94]. Biofilms are constituted from clusters of microorganisms held together in a selfproduced matrix made of proteins, polysaccharides, and environmental DNA (eDNA). Protected within the biofilm environment, pathogenic bacteria become a growing threat for human health, especially in the case of chronic infections. The biofilm matrix promotes bacterial infections by improving colonies’ adhesion to surfaces by creating a favourable microenvironment for bacteria to grow and by physically shielding bacterial cells from the immune system and the action of antibiotics [95]. Owing to their small size, CD nanoparticles have been shown to be able to penetrate biofilm matrices and eradicate or prevent its formation in both Gram-positive and Gram-negative bacteria both achieving broad antibiofilm action [78], as well as specific targeting towards Gram-positive [55,96] or Gram-negative [97] bacteria. Otis et al. [98] recently prepared aminoguanidine/citric acid-based CDs able to selectively label Gram-negative *P. aeruginosa* (but not *S. aureus*) and to inhibit *P. aeruginosa* biofilm formation. The authors found a correlation between the N-content doping and the bactericidal effect against *P. aeruginosa.* Similarly, CDs prepared from *Artemisia argyi* leaves could selectively inhibit *E. coli* over *S. aureus* biofilm formation, promoting cell death via membrane intercalation and damage [99]. 

**Figure 5:**
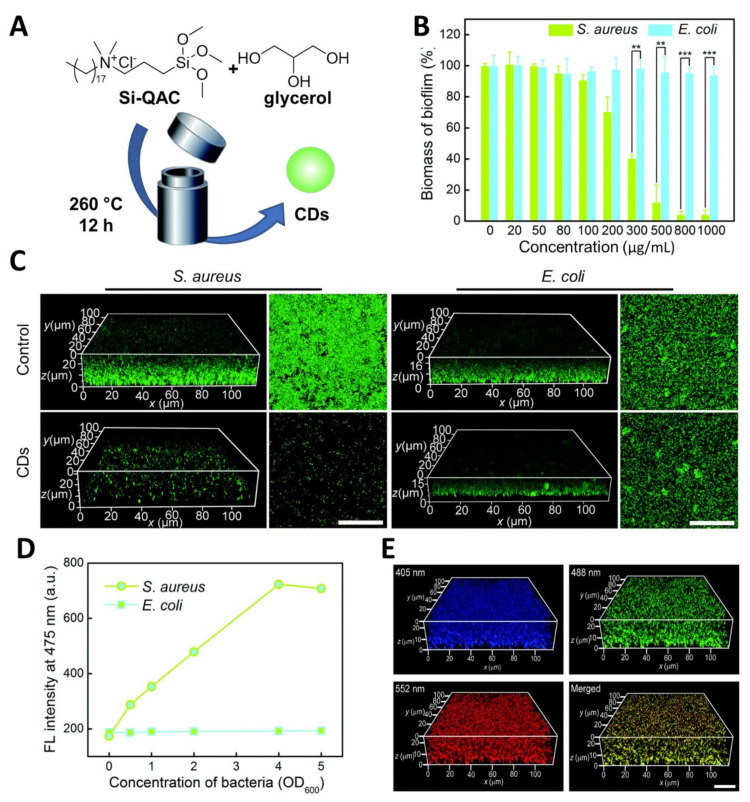
(**A**) General synthetic approach for the solvothermal synthesis of fluorescent CDs. (**B**) Change of biofilm biomass as a function of CD concentration. (**C**) 3D and 2D confocal fluorescence images of *S. aureus* and *E. coli* biofilms formed by the untreated (control) and CD (50 μg/mL)-treated *S. aureus* and *E. coli* bacteria. Before imaging, the biofilms were stained with SYTO 9. Scale bars: 30 μm. (**D**) Fluorescence (FL) intensity of CDs (6 mg/mL) in the presence of *E. coli* and *S. aureus* at different bacterial concentrations (OD_600_ = 0.0–5.0). (**E**) 3D confocal fluorescence images at different excitation wavelengths (405, 488 and 552 nm) of *S. aureus* biofilm stained with CDs (50 mg/mL) for 2 h. Scale bar: 20 mm. Reproduced with the permission from RSC [55].

In a different study, Wang et al. [56] reported the use of quaternary ammonium salts as CD precursors, showing that these strongly positively charged nanoparticles exerted a stronger bactericidal effect on *S. aureus* over *E. coli* and were able to eradicate *S. aureus* biofilm via membrane damage and ROS formation. These nanoparticles accelerated in vivo wound healing in mice models, achieving better results on methicillin-resistant *S. aureus* compared to commonly used antibiotics such as penicillin and vancomycin. The Wu research group found further applications for the previously described Si-QAC CDs used for the selective labelling and killing of Gram-positive bacteria [91]. The new modified version of the Si-QAC CDs found further applications in biofilm fluorescent labelling and eradication [55]. The group prepared the novel CDs through a solvothermal synthesis of Si-QAC and glycerol in a 1:2 ratio (Figure 5A). These CDs could stain both *E. Coli* and *S. aureus* biofilms (Figure 5C) promoting selective *S. aureus* biofilm eradication at a CD concentration of around 1 mg/mL (Figure 5B). Furthermore, the authors demonstrated the capability of the CDs to inhibit *S. aureus* biofilm formation at a concentration of 50 µg/mL (Figure 5C). The selectivity for *S. aureus* labelling was further confirmed by measuring the fluorescence intensity of CDs incubated with different concentration of *E. coli* and *S. aureus*, showing negligible labelling for *E. coli* (Figure 5D).

Another interesting property of CDs is the excitation-dependent emission of these nanoparticles [100,101]. This type of emission profile can be quite versatile since the CDs can emit in different regions of the spectrum depending on the chosen excitation wavelength, thus allowing for the simultaneous acquisition of fluorescent images from the same sample at different wavelengths. The authors managed to acquire multicolour fluorescent images *S. aureus* biofilm under excitation at 405, 488, and 552 nm (Figure 5E). Finally, they demonstrated via changes on the ζ-potential measurements of the bacteria/CD adducts that the positively charged CDs strongly bind to *S. aureus* rather than *E. coli*, and that biofilm eradication via membrane disruption-promoted antibacterial effects.

Overall, it was demonstrated that CDs can be efficiently used for antibiofilm applications, demonstrating promising bactericidal and staining properties of these carbon-based materials or biofilm inhibition and imaging, paving the way for the use of CDs in the treatment of biofilm infections.

### 4.3. Applications of CDs as Drug Delivery Carriers

Whereas CDs alone showed great antibacterial and antibiofilm activity, these carbon-based nanomaterials can also be used as scaffolds for drug delivery applications. By taking advantage of the greater cellular internalization of these nanoparticles, CDs can be used as vectors to enhance drug uptake through the cell membrane. This feature found direct use for the delivery of antibiotic molecules inside bacterial cells and for the clearance of bacteria living inside host eukaryote cells. In some examples, antibiotics such as gentamicin sulphate and ampicillin (AMP) were directly used as precursors for the synthesis of CDs via solvothermal methods [88,102]. This strategy furnished CDs that displayed on their surface entire or active fragments of the antibiotic precursor used as starting material, providing a nanomaterial capable of killing bacteria through combined antibiotic delivery, membrane damage and photodynamic effects. Nonetheless, in most reported examples, CDs are produced from cheaper carbon sources and their surface is typically postfunctionalized with antibacterial agents either via covalent or noncovalent ligation techniques [103,104], and some of the key examples will be discussed below.

Recently, the Gedanken group developed *m*-phenylenediamine-derived CDs via hydrothermal treatment that exhibit antibacterial activity at concentrations of 1 mg/mL [46]. To improve the antibacterial action of the CDs, the nanoparticles were loaded with ciprofloxacin via an H-bond and electrostatic interactions by simple stirring a mixture of the CDs and the antibiotic in water. A high drug loading (60% *w*/*w*) was achieved in the nanocomplex with a pH-dependent drug release. At a pH of 5.0, 90% of ciprofloxacin was released within 5 min, while at physiological pH 7.4 the system reached a 40% drug release after 38 h. In a subsequent study, the same group reported the use of waste jute-derived quaternary CDs complexed with ciprofloxacin bound via similar H-bond and electrostatic interactions [105]. This complex also showed high drug loading (62% *w*/*w*), similarly to the previously described phenylenediamine-based CDs and demonstrated that the nanocomposite could eradicate both *E. coli* and *S. aureus* at very low concentrations (0.48 µg/mL and 0.03 µg/mL, respectively). The authors hypothesized that the initial CD–bacteria interaction was due to electrostatic attraction between the positively charged CDs and the negatively charged bacterial surface. Subsequently, the group demonstrated using electron paramagnetic resonance measurements that the antibacterial mechanism of action was attributed to the formation of ROS, leading to subsequent membrane and DNA damage.

**Figure 6:**
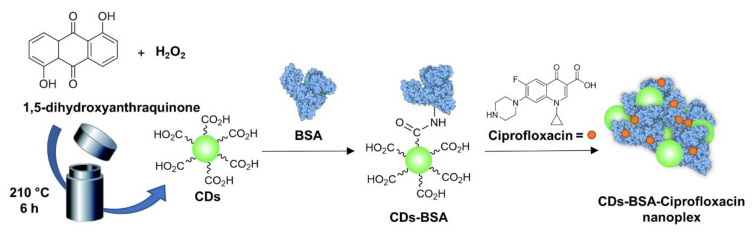
General schematic approach for the solvochemical synthesis of fluorescent CDs, BSA conjugation, and Ciprofloxacin complexation [106].

Mandal et al. [106] also used ciprofloxacin which was loaded on green fluorescent 1,5-dihydroxyanthraquinone-based CDs produced via a solvothermal method (Figure 6). In this example, bovine serum albumin (BSA) was used to coat the CD surface via EDC-promoted amidation reaction, and they found that the BSA coating improved the CD ROS production activity. The authors postulated that the protein could act as a shield, protecting the active functional groups present on the CD surface from oxidative degradation. Ciprofloxacin was then complexed via noncovalent interactions with the CD-BSA conjugate, furnishing a drug nanoplex. The system exploited the synergistic effects of photodynamic ROS production and antibiotic drug release leading to a 95% killing of both *E. coli* and *S. aureus* at concentrations as low as 1.47 µg/mL of the CD-BSA-ciprofloxacin complex. 

**Figure 7:**
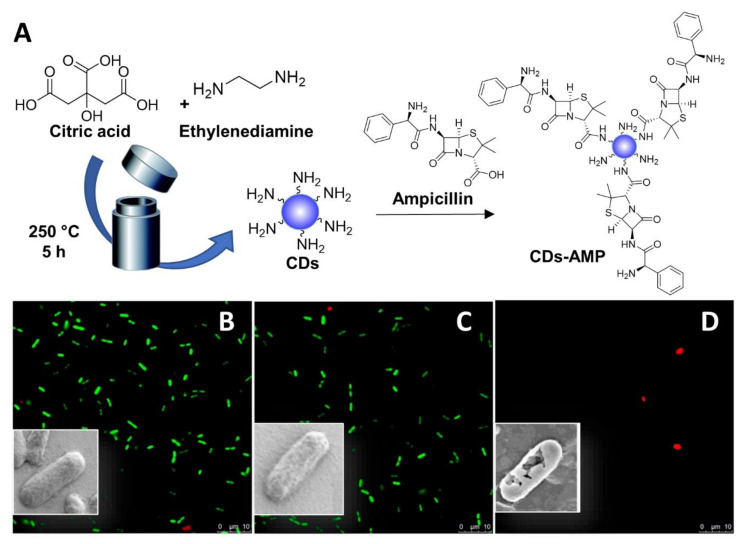
General synthetic approach for the solvothermal synthesis of fluorescent CDs and AMP functionalization (**A**). Viability of *E. coli* K12-MG 1655 imaged by the LIVE/DEAD BacLight Bacteria images of: control without CDs (**B**); with CDs (100 μg/mL) (**C**); with CD-AMP (100 μg/mL) (**D**). Inserts: SEM images of bacterial cell. Reproduced with the permission from Elsevier [107].

In addition to noncovalent drug CD-conjugates, covalently attached CD-antibiotic complexes have also been described. For instance, Jijie et al. [107] reported the functionalization of citric acid-based CDs with AMP via EDC-promoted amide coupling (Figure 7A). The CD-AMP conjugates exerted a stronger antibacterial activity compared to AMP alone owing to the superior cellular permeabilization properties of the CD scaffold, leading to improved drug internalization inside bacterial cells. While CDs alone did not have any effect on *E. coli*, fluorescent and SEM imaging showed membrane damage in CD-AMP treated bacteria (Figure 7B–D). Interestingly, while the lowest concentration of AMP necessary to inhibit the visible growth of *E. coli* was 25 µg/mL, only 14 µg/mL was required when using AMP-CDs. Furthermore, *E. coli* cells treated with CDs and CD-AMP conjugates at 400 µg/mL showed a significant decrease in viability after visible sun light irradiation for both conjugated and unconjugated nanoparticles, albeit with an enhanced bactericidal effect for CDs-AMP. The authors demonstrated that the CD-AMP conjugates combine the antimicrobial activity of AMP with the ability of the CDs to promote bacterial membrane damage and photodynamic ROS formation. 

**Figure 8:**
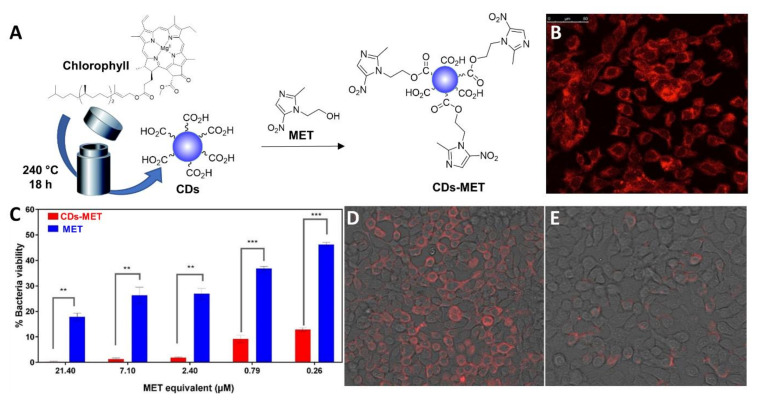
(**A**) General synthetic approach for the solvothermal synthesis of fluorescent MET-functionalized CDs. (**B**) Fluorescence images showing cellular uptake of CD-MET after 4 h. (**C**) *P. gingivalis* colonies numbers (CFU) from blood agar cultures after treatment with MET and CD-MET conjugates. ** = (*p* ≤ 0.01) and *** = (*p* ≤ 0.001). (**D**) *P. gingivalis* labelled cells with a specific antibody in the red channel and treated with MET at a concentration of 7 µM. (**E**) *P. gingivalis* labelled cells with a specific antibody in the red channel treated with 7 µM of CD-MET (MET-equivalent). Reproduced with permission from Elsevier [108].

In another elegant example, Ardekani et al. [108] reported the synthesis of CDs from chlorophyll as the starting material under hydrothermal conditions and their functionalization with metronidazole (MET) as the antibiotic via a combination of ester conjugation and H-bond interactions (Figure 8A). The CD-MET conjugates contained a very high drug loading up to 80% (*w*/*w*). The nanoparticles exhibited a 90% cellular internalization after 3 h incubation with H413 oral epithelial cells (Figure 8B). Studies against *Porphyromonas gingivalis* bacterial cells showed that the CD-MET conjugates were 3-fold more active compared to free MET in reducing the bacterial viability inside host cells (Figure 8C). Moreover, the CD-MET conjugates were internalized at higher levels than MET; as a result, the CD-MET conjugates could completely eradicate intracellular *P. gingivalis* with an equivalent MET concentration as low as 7 µM (Figure 8D,E).

The use of CD–antibiotic conjugates furnished improved antibacterial activity in many instances when compared to the free drug, owing to a combination of synergic bactericidal effects provided by the antibiotic drug and the CD photodynamic inactivation of bacteria. Moreover, CD–antibiotic conjugates have shown great antibacterial activity even in pathogens that are known to be extremely difficult to eradicate thanks to the better targeting and internalization efficiency of the CD platforms in bacterial cells. 

## 5. CD-Based Composite Materials for Antibacterial Applications

CDs have also found wide applications in the preparation of novel composites as part of hybrid materials [109]. Composite materials combine the properties of different nanostructures; for instance, CDs can be embedded into solid polymer structures. In this manner, the antibacterial properties of the CDs can be supported on a solid phase, facilitating the removal or washing of the composite [110]. Many bactericidal applications of CD-composites rely on combining the photodynamic ROS production of the CDs with the antibiotic activity of a transition metal-based nanoparticle [111], often containing Ag [84], Cu [112,113,114], Zn [115,116,117], or Ti [118] oxides cores, among others. Moreover, CDs have been embedded within polymeric structures, including hydrogels and polymers of different natures, ranging from chitosan [119,120] to DNA [121], cellulose [122], gelatin [123], polyurethane [124], or poly lactic-co-glycolic acid nanoparticle [125] matrices. These composites have found ample antibacterial applications (such as in water treatment [126,127,128,129] and the surface coating field [130,131,132]). In many cases, the CD-composites showed bactericidal activity upon sun light illumination [133,134,135,136]. It is worth noting that the use of metal-based composites for antibacterial applications may be counterbalanced by the inherent environmental and eukaryotic cellular toxicity of transition metals derivatives that should be taken into account to exclude off target effects [137,138,139,140].

**Figure 9:**
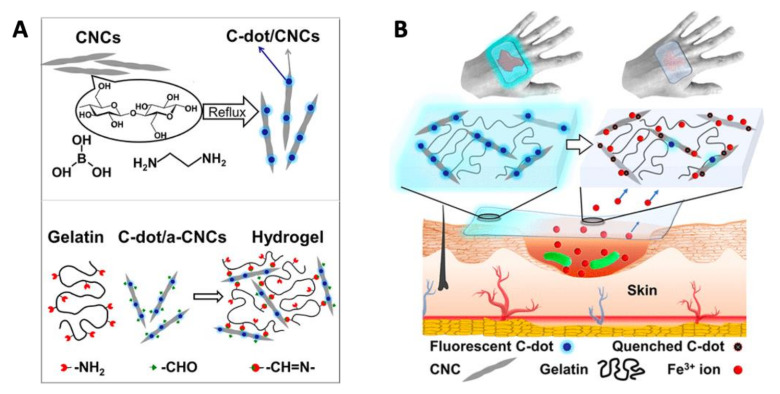
(**A**) General synthetic approach for the hydrothermal synthesis of fluorescent CD/cellulose nanocrystals-based hydrogel. (**B**) Schematic representation of CD/cellulose nanocrystals-based hydrogel performance as a wound dressing depriving the wound of Fe^3+^ ions. Reproduced with permission from ACS [138].

Apart from ROS generation and membrane damage, Chekini et al. [141] synthesized a CD/cellulose nanocrystal-based hydrogel that was able to sequester Fe^3+^ from the surrounding environment. In this manner, bacterial cells were depraved of cationic iron, which is essential for bacterial growth and reproduction and, thus, were inhibited in the growth of both Gram-positive and negative bacteria. The CDs were prepared from cellulose nanocrystals via hydrothermal preparation and then crosslinked with aldehyde-modified cellulose to form the hydrogel (Figure 9A). The material showed a complexation-derived fluorescence quench upon coordination with the metal, which is useful to determine the effectiveness of the material by the naked eye (e.g., the absence of fluorescence correlates with the inactivation of the material and therefore must be replaced to sequester more iron (Figure 9B)). The antibacterial and fluorescent properties of this material combined with the biocompatibility with human cell lines and the possibility to extrude the hydrogel using a 3D printer makes these types of composites optimum “smart” material for wound dressing applications.

**Figure 10:**
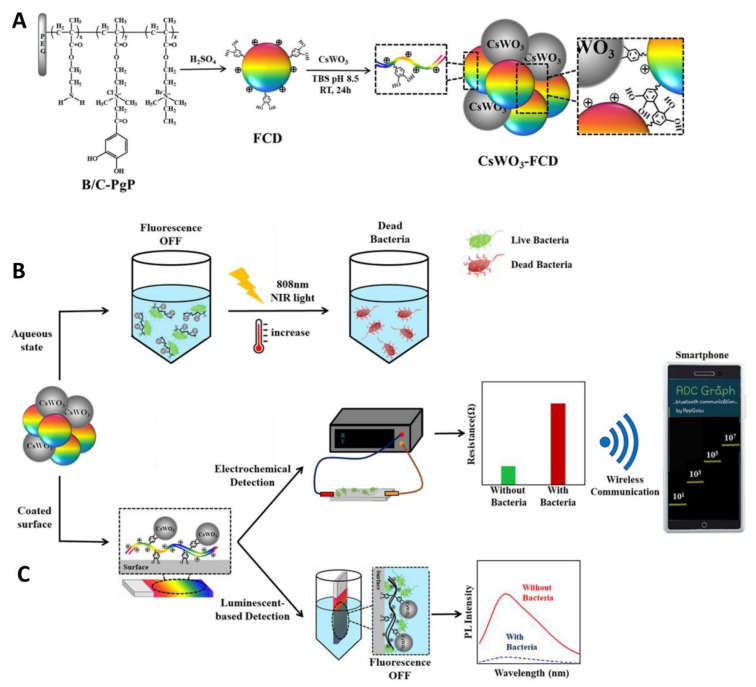
(**A**) Schematic illustration of CD–CsWO_3_ nanohybrid fabrication. (**B**) Fluorescent detection and photothermal killing of bacterial cells with CD–CsWO_3_. (**C**) Schematic representation of CDs–CsWO_3_ nanohybrids preparation on solid phase for wireless electrochemical- and luminescent-based bacterial sensing and applications on the photothermal ablation of bacteria. Reproduced with permission from Elsevier [144].

As previously discussed, CDs drew great attention owing to their possible applications in biomedical devices. The remarkable fluorescent properties of CDs (including chemical stability, low degree of photobleaching, and great human biocompatibility) make them excellent candidates for the sensitive detection of pathogenic bacteria [50,142]. To this extent, Park and coworkers reported the synthesis of CD nanoparticles starting from a quaternarized ammonium polymer prepared via reflux under acidic conditions. In a first study, the authors prepared a composite between catechol-functionalized CDs and Ag nanoparticles [143]. The fluorescence of the CD–Ag composites was quenched after interacting with the bacterial surface and this could be exploited for the successful fluorescence-based identification of bacteria in contaminated river waters. In a follow-up study, the same group prepared CD–CsWO_3_ nanocomposites for both fluorescent and electrochemical identification of bacteria built using the same catechol-promoted complexation (Figure 10A) [144]. The blue fluorescent CDs–CsWO_3_ nanohybrids were quenched upon binding to the surface of *E. coli* and *S. aureus* (Figure 8B). This luminescent-based bacterial detection system could be used to detect bacteria in solution with a limit of detection of 70 and 131 CFU/mL for *E. coli* and *S. aureus*, respectively. Moreover, taking advantage of the photothermal properties of CsWO_3_ nanoparticles, the authors showed complete bacterial killing via membrane disruption processes, promoted by NIR irradiation at 808 nm (Figure 10B). The team further improved the sensitivity of this technique using the CD–CsWO_3_ composite to coat a Si wafer surface, creating a probe that could simultaneously detect and kill bacteria in the solid state (Figure 10C). The electrochemical sensor measured differences in resistance that occurs at the sensor surface after interaction between the cationic moieties of the nanohybrid-coated sensor and the negatively charged bacterial cell wall. The system was wirelessly connected to a smartphone, allowing for the streamlined detection of bacteria with an impressive limit of detection <10 CFU/mL for both *E. coli* and *S. aureus* (Figure 10C).

## 6. CD-Based Functional Materials for In Vivo Biomedical Applications

The treatment of MDR bacterial infections is a major challenge for hospitalized patients, especially in the case of chronic infections, which translates into a direct threat for patient’s life and drastically increases the sanitation and hospitalization costs and patient recovery time. Owing to the CDs’ excellent biocompatibility and great bactericidal performances, the implementation of CDs in novel biomedical materials constitutes a promising approach to suppress MDR bacterial infections in the clinical setting and as components in pharmaceutical and wound dressing materials. These novel materials possess great potential for the topological treatment of infections, inhibiting bacteria and biofilm growth while at the same time delivering a long-lasting protection against pathogens.

**Figure 11:**
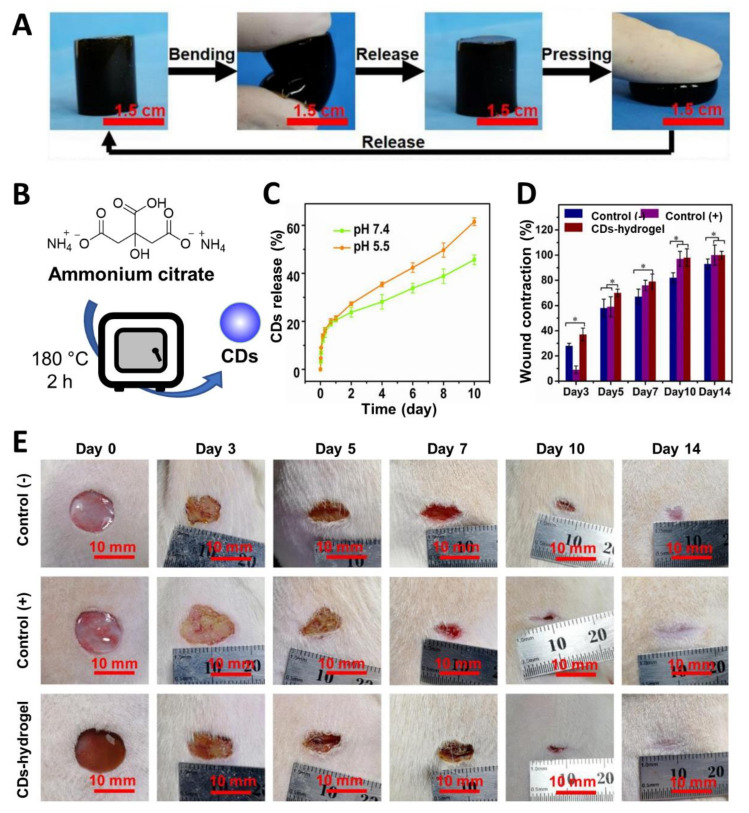
(**A**) Soft and flexible CD–hydrogel materials. (**B**) General synthetic approach for the synthesis of CDs from ammonium citrate. (**C**) CD release curves at different times at pH 7.4 and 5.5 in PBS. (**D**) Wound contraction for the three hydrogels treatment. * *p* < 0.05. (**E**) Wound healing activity overtime upon treatment with CD–hydrogels. Commercial hydrogel used as (+) control and hydrogel–CD without gentamicin functionalization used as (−) control. Reproduced with permission from Elsevier [145].

Recently, Li et al. [145] prepared ammonium citrate-based CDs via thermal decomposition at 180 °C in a muffle furnace (Figure 11B). Afterwards, the CD surface was functionalized with gentamicin through a calcination process at 180 °C. The nanomaterials were complexed with carboxymethyl chitosan to provide a CD–chitosan composite. Then, the free amine functions of the chitosan were further reacted with aldehydes present on a dextran polymer derivative to furnish the final hydrogel via imine formation. The CD-containing hydrogel presented good physical flexibility (which is ideal for its use on the irregular shape of wounds (Figure 11A)). Moreover, the hydrogel showed a pH-dependent release of the CDs owing to its ability to cleave imine bonds under acidic pH (Figure 11C). As already seen in other studies, this pH-responsive behaviour is useful for the treatment of biofilm infections that are usually associated with an acidic microenvironment [146]. Studies against *E. coli* and *S. aureus* bacteria revealed good antimicrobial and antibiofilm properties with low drug resistance for gentamicin/CD-functionalized hydrogels. Lastly, in vivo assays on wound healing models conducted on the back of rats infected with *S. aureus* revealed better skin healing capability for the drug/CD–hydrogel compared to commercially available hydrogels used as positive controls (Figure 11D,E) and showed good biocompatibility and wound healing properties, which allowed for faster wound closure and long-term protection throughout the whole recovery process against *S. aureus* infections.

Another interesting example of CD/hydrogels composites for wound healing applications was reported by Xiang et al. [147]. In this study, the authors reported the synthesis of a ZnO/CDs hydrogel nanocomposite that uses folic acid/dopamine hydrogels, which are stabilised via complexation with Zn^2+^. The system exhibited antimicrobial properties and tissue repair abilities; moreover, the presence of Zn and CD nanoparticles also allowed for PTT activation [148]. Indeed, the application of the nanomaterial improved fibroblast growth, leading to accelerated wound repair and long-lasting antibacterial effects via both PTT and ROS generation under dual illumination at 660 and 808 nm. Rat wounds infected with *S. aureus* and treated with the ZnO/CD hydrogel showed a wound closure rate of 95.7% after 10 days compared with a rate of 70.5% for the control group treated with just PBS.

The use of transition metals within the CD structure may confer better antibacterial properties to CD-based materials. However, for in vivo applications, these type of materials need to undergo extensive animal testing to ensure the absence of toxic side effects that are in many cases associated with transition metal nanoparticles [138,139,141]. To address this, Tang et al. [149] prepared a metal-free photocatalyst based on CDs and graphitic carbon nitride (g-C_3_N_4_) capable of eradicating *S. aureus* infections under visible light irradiation. The nanoconstruct produced an enhanced ROS-promoted antibacterial effect, superior to those of CDs and g-C_3_N_4_ alone, with no antimicrobial activity found in the absence of light irradiation. Moreover, tests in mice models infected with *S. aureus* revealed an enhanced wound regeneration effect for treated mice in the presence of visible light, while no difference was observed in the absence of light.

**Figure 12:**
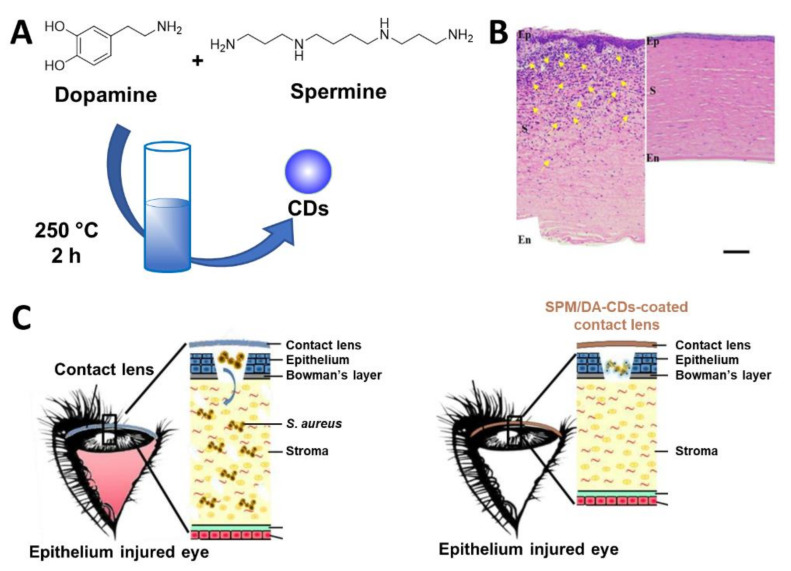
(**A**) General synthetic approach for the synthesis of SPM/DA-CDs. (**B**) Histological examination of corneal tissue sections by staining with hematoxylin, eosin, and Gram. Yellow arrows indicate inflammatory cell infiltration. Ep: epithelium; S: stroma; En: endothelium. Scale bar indicates 100 μm. (**C**) Representation of the mechanism of action of SPM/DA-CDs-coated contact lenses used to alleviate *S. aureus* bacterial keratitis development in an injured cornea. Reproduced with permission from Elsevier [152].

CDs can also be embedded inside patches for wound dressing purposes or inside nanofibrous materials commonly used in biomedical devices for a slow release and long-term antibacterial protection [150,151]. For instance, Jian et al. [152] showed that spermidine/dopamin-based CDs (SPM/DA-CDs) can be used for the functionalization of 2-hydroxyethyl methacrylate, a polymer film that constitutes the main component of contact lenses. The CDs were prepared via hydrothermal degradation of a SPM/DA mixtures at 250 °C for 2 h (Figure 12A). The new CDs inhibited the growth against a range of different bacteria, including *E. coli* and *S. aureus* at a concentration of 10 µg/mL. Soaking of the 2-hydroxyethyl methacrylate film in a solution containing the SPM/DA-CDs at 200 µg/mL furnished CDs-functionalized contact lenses used for in vivo assays. The trials implied a modified keratitis model using *S. aureus* to infect the scratched cornea of rabbit eyes. Rabbit treated with SPM/DA-CD contact lenses revealed a faster recovery over the untreated control group, restoring a clear cornea as in normal eyes (Figure 12C). The recovery was also confirmed by reduced overall inflammation after 3 days and reduced corneal thickening for rabbits treated with SPM/DA contact lenses (Figure 12B).

## 7. Conclusions

The emergence of antimicrobial resistance represents a significant health and economic challenge worldwide. The slow pace of antibacterial discovery requires the development of novel antimicrobial drugs, but it also requires the development of improved strategies for the repurposing of existing agents and effective diagnostic tools that can inform antibiotic prescription. In recent years, the development of novel probes able to target bacteria for detection and killing as effective theranostic strategies has grown into a focus of great importance. Carbon dots (CDs) have emerged as promising bioimaging probes due to their many advantages over molecular fluorophores and other fluorescent nanoparticles, since the preparation of these water-soluble carbon-based nanomaterials is often practical and low-cost. These materials exhibit amenable physicochemical properties such chemical and photochemical stability and low toxicity, which makes them ideal for biological applications, as we have seen in the examples described herein. Indeed, since their discovery, this class of carbon-based fluorescent nanodots have found many applications in the biological and biomedical arena. Since the CD’s functional and biological properties are linked to the nanomaterial molecular structure which is dependent on the choice of starting materials and synthetic strategy, it has become evident that in order to develop tailored carbon-based nanomaterials for bespoke applications, more efforts are still needed to define the key parameters required to develop robust and reproducible synthetic strategies that lead to homogeneous materials with defined molecular features. Not surprisingly, we now appreciate that not all carbon dots are the same and that their structural features (e.g., type of core and surface functionalities) have a significant effect on how these materials interact with other cells and organisms, and this can be exploited to design probes that can target specific bacterial classes (such as Gram-negative or Gram-positive bacteria). In addition to their fluorescent properties being use as a means to label and image bacteria, CDs have been found to act as photosensitisers that can be activated with excellent spatiotemporal control upon irradiation with light, leading to the production of ROS and membrane damage. Nanocomposites of antimicrobial agents (e.g., antibiotics and transition metals) with CDs have also been shown to be effective theranostic agents with improved antimicrobial activities that combine the synergistic effects of their individual components. Moreover, CDs have also found applications in the development of novel materials such as hydrogels, nanofibrous materials, and polymers for would healing and regeneration applications, as well as in materials that can provide long-lasting protection against infections.

As we gain a better understanding at a molecular level of the key functional parameters required for bacterial targeting, improved and specific CD-based probes and materials will be designed. This is a rapidly growing area of research that has shown tremendous advancements already, and we look forward to following the progress in the coming years.

## Data Availability

Not applicable.
